# Quantitative analysis of transversal dentofacial asymmetries using combined 3D jaw and face-scans: a prospective analytical cross-sectional study

**DOI:** 10.1186/s13005-026-00617-x

**Published:** 2026-03-28

**Authors:** Johanna Radeke, Franziska Lara Brand, Fatih Kilic, Rudolf Jäger, Stefan Repky, Bernd Georg Lapatki

**Affiliations:** 1 Department of Orthodontics, Ulm University, Ulm, Germany; 2 Institute of Epidemiology and Medical Biometry, Ulm University, Ulm, Germany

**Keywords:** Three-dimensional scans, Dentofacial asymmetry, Face imaging, Jaw imaging, Dentofacial deviations, Dental midline, Dentofacial esthetics

## Abstract

**Background:**

The spatial position of the dentition relative to the perioral soft tissues is predominantly documented and evaluated two-dimensionally using cephalometric or photographic methods. However, objective quantitative assessment is difficult with two-dimensional imaging modalities, especially in cases of transverse asymmetries. The aim of this study was to assess the potential of three-dimensional (3D) face-jaw imaging and analysis of transverse deviations using combined 3D surface scans.

**Methods:**

Combined 3D data of the face and jaws of 76 untreated subjects were evaluated. Group A (*n* = 66) included patients with no evidence of tooth migration beyond the maxillary dental midline (dentMid), e.g., due to premature loss of primary teeth. The patients, whose imaging data showed tooth migration, were assigned to group B (*n* = 10). All 3D models were evaluated three times each by four examiners, and analysis parameters relevant to facial and dentofacial asymmetries were calculated and described. “True” landmark positions were estimated using a linear mixed-effects model. Variability of relevant results was characterized by standard deviations which were combined using a linear model. For group A, the relationship between the deviations of the dentMid and of the skeletal maxillary midline (maxMP) from the facial midline plane (facMP) was statistically evaluated by calculating the Spearman correlation coefficient. The difference between these deviations was analyzed using the Wilcoxon test.

**Results:**

In group A, a median difference of only − 0.4 mm (range: -1.4 – +0.7 mm) was observed between dentMid and maxMP. Corresponding differences for patients assigned to group B were − 0.6 mm (-1.9 – +0.4 mm). The Spearman correlation analysis indicated a strong correlation between the two parameters (𝑟 = 0.84) and the Wilcoxon test indicated no significant difference between these deviations (*p* = 0.995).

**Conclusions:**

Our quantitative analysis of combined 3D surface scans demonstrates the wide range of the differences between dental and skeletal midline deviations in untreated individuals. The possibility to differentiate skeletal and dental aspects of midline deviations through an approach without X-rays is of particular therapeutical relevance in patients with pronounced skeletal midline deviation, to quantitatively assess the amount of dentoalveolar compensation required to align the dentMid to the facMP.

## Background

Orthodontic procedures refer to clinical treatments used to correct a wide range of anomalies of both tooth and jaw positions. A possible side effect of these procedures is that they may affect the surrounding facial soft tissues. For example, changes in the axial position of the anterior teeth can lead to padding or flattening of the lip profile and can be decisive in the decision for or against extraction therapy [1–3]. Furthermore, procedures such as mandibular protrusion or retrusion and maxillary advancement – in extreme cases by orthognathic surgery – can result in profile changes [4, 5]. In addition to the sagittal aspect of the profile, also the frontal view has a significant aesthetic influence. The corresponding tooth-soft tissue relationships include both vertical aspects (e.g., the inclination of the occlusal plane), and transverse aspects (e.g., the relationship of the dental midline to the facial midline). The latter relation plays an important role in patients in which the maxillary midline and the median palatine raphe respectively do not coincide with the facial midline. In these patients, therapeutic dentoalveolar compensation of the deviation between the facial and dental midlines is necessary, which may have implications for the analysis of space requirements from a diagnostic point of view. Accordingly, a major goal of orthodontic diagnostics is the quantitative determination of the positional relationship between the dentoalveolar complex and the facial soft tissues.

While the use of digital, three-dimensional (3D) jaw models is already clinical standard, the spatial position of the dentofacial structures (i.e., the teeth, jaws, and perioral soft tissues) relative to one another has been predominantly documented and evaluated two-dimensionally using cephalometric and photographic methods. However, two-dimensional imaging is limited by parallax and alignment errors, as well as the availability of only standardized views, which may obscure some surfaces and, thus, not reflect all problem areas and may not provide quantitative measurements at all. Three-dimensional (3D) radiographic images such as CT or cone-beam CT (CBCT) scans are taken if necessary, but for radiation safety reasons, these are not part of standard orthodontic diagnostics [6]. Given this situation, 3D scans of the facial surface offer an X-ray-free alternative for documenting facial soft tissue without such limitations. This method may be a valuable addition to the diagnostic toolbox due to its ability to provide a wide range of views and 3D digital evaluation. Furthermore, the integration of 3D jaw and facial scans offers the possibility of a 3D facial representation of jaw models without the use of X-rays [7–11] and might be a promising avenue for the detection and quantification of dentofacial asymmetries [12, 13].

As aforementioned, an anterior dental midline shift in the maxillary arch is a frequently occurring and aesthetically relevant transverse dentofacial asymmetry [14] which is usually attributed to a dental cause [15, 16]. They tend to occur after premature loss of primary teeth, undermining resorption, or trauma. However, in some patients with such dental maxillary midline shifts, there are no visible signs of tooth migration or asymmetries in the dental arch. A logical conclusion is that the existing deviations of the dental maxillary midline from the facial midline in these patients are caused by other factors, such as skeletal asymmetry of the maxillary complex. Such cases would particularly benefit from 3D analysis of combined jaw-facial scans, which could help objectively and quantitatively assess underlying causes such as deviations (in the form of rotations or shifts) of the raphe median plane in relation to the facial midline plane.

The aim of this study was to explore the potential of X-ray-free 3D combined jaw-facial scans in the differential diagnosis of transverse deviations of the maxillary dental midline. For this purpose, the frequency, severity and differential diagnosis of such midline deviations and other facial transverse asymmetries were examined in a patient group that has not yet undergone orthodontic treatment. To this end, 3D analyses comprising analyses of transversal facial and dentofacial aspects were developed, and their potential usefulness in routine orthodontic diagnostics were explored.

## Methods

### Study participants

Patients who had all the teeth necessary for the corresponding evaluation and complete facial and jaw model scans were included. The following criteria had to be met for inclusion:

Upper jaw:

- Canines (permanent or deciduous) completely erupted; if not present or incompletely erupted, the first premolar or deciduous molar on that side had to be completely erupted.
- Premolars/deciduous molars completely erupted; if not present or incompletely erupted, the canine (deciduous or permanent) and second premolar/deciduous molar on that side had to be completely erupted.
- Permanent central incisors and first molars completely erupted.
- The structures of the palate had to be completely represented.

Patients with craniofacial malformations, multiple dental aplasias (more than one tooth per quadrant), previous orthodontic treatment, or fixed orthodontic appliances were excluded. Based on these criteria, 76 of 100 consecutive patients aged 6 to 67 years admitted to the Department of Orthodontics and Dentofacial Orthopedics at Ulm University Hospital were included. The study was conducted in accordance with the declaration of Helsinki and has been approved by the Ethics Committee of Ulm University Hospital (No. 300/18). All participants gave their written consent for the use of their data.

The study cohort was divided into two groups: group A (*n* = 66) consisted of patients without signs of tooth migration beyond the midline of the maxilla (e.g., due to premature loss of primary teeth or undermining resorption) and group B (*n* = 10) included patients who showed clear signs of such tooth migration. The patients were grouped based on digital imaging models by an orthodontist who was blinded to the study aims and protocols and was not involved in the study.

### Face scans

A structured light scanner (optoTOP-HE; Breuckmann GmbH, Meersburg, Germany) was used to capture 3D images of the face. The optoTOP-HE offers high measurement accuracy of 2 μm and resolution (1.3 megapixels) and was used in previous anthropological and medical research [17–24]. The facial scans were taken with the participant sitting upright with a natural head posture, relaxed facial muscles, and closed lips. Participants were asked to “bring their posterior teeth in contact with minimal muscular effort”. In this manner, we aimed at achieving habitual occlusion without clenching in order to avoid changes in facial contours due to increased masticatory muscle activity. To visualize the entire face, including the auricles, the facial surface was scanned from both the frontal and oblique lateral (45°) angles on the right and left sides. For an additional frontal scan, the participant’s frontal teeth were matte-coated with anti-reflective spray (BlueSpray; Devemed, Neuhausen ob Eck/Tuttlingen, Germany), and the face and frontal teeth were scanned simultaneously, with the lips held away from the teeth using lip retractors [11, 25].

### Digital dental arch models

Digital dental arch models were indirectly produced by first creating plaster models (Type III hard stone) from impressions of the jaws made with alginate. These models were allowed to set and dry for at least 24 h, following which they were digitized using the desktop scanners dStation3D (Breuckmann GmbH, Meersburg, Germany) or R2000 (3Shape, Copenhagen, Denmark).

### Merging and registration of the scans

The Optocat software (release 15R2; Breuckmann GmbH, Meersburg, Germany) was used to merge the resting face scans taken from the three different perspectives and to register the digital jaw models to the facial surface. The scanned surfaces were superposed using the best-fit method included in Optocat.

**Fig. 1:**
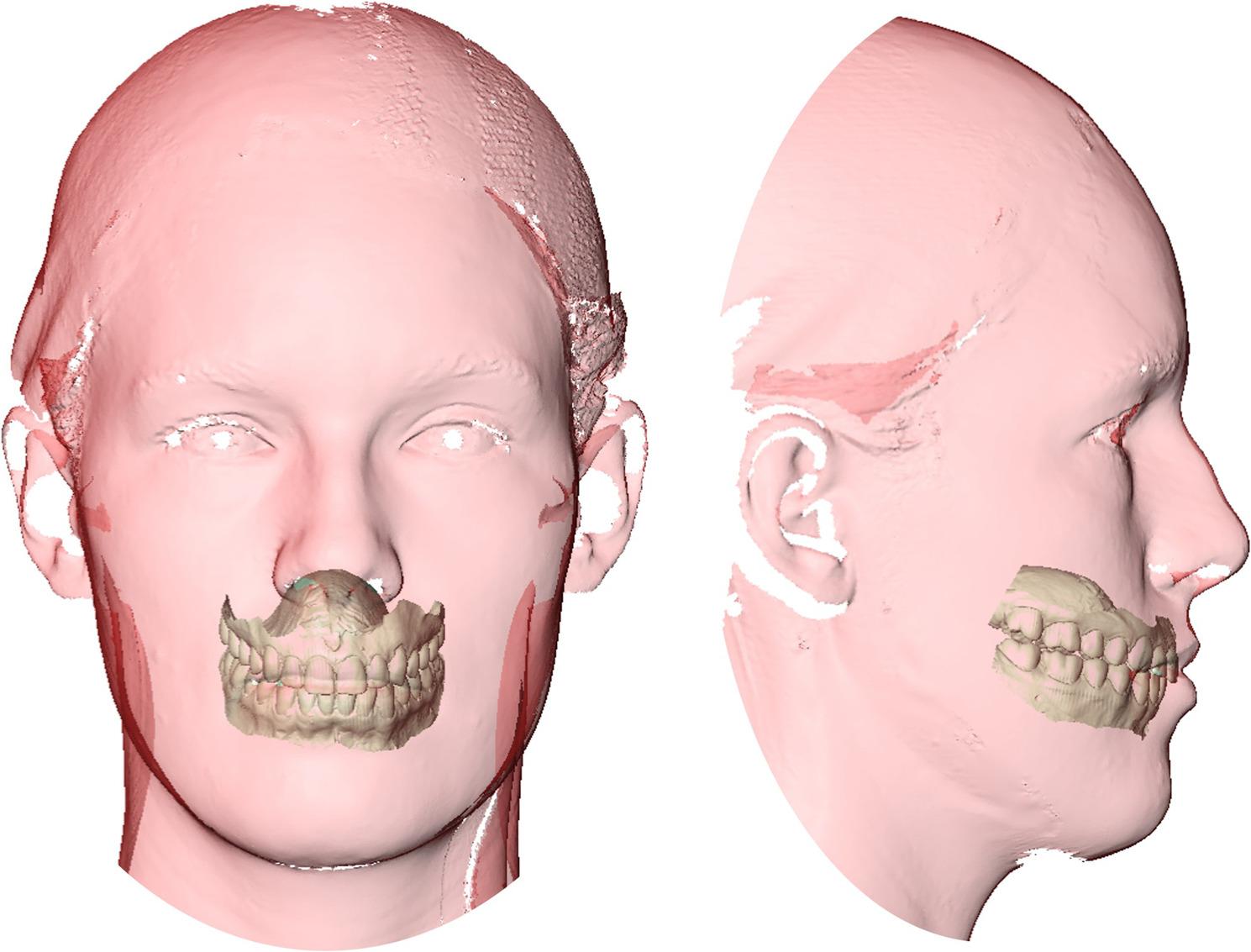
Integrated 3D jaw-facial scans

The face scan with retracted lips was utilized as a registration template. First, the complete resting scan was registered to the extended lips scan by superposing shape- and position-stable areas on the forehead, temples, and nasal bridge, with the maximum distance set at 0.1 mm. Next, the maxillary jaw model was registered on the scan with retracted lips by superposing the labial surfaces of the upper anterior teeth. The mandibular arch model was spatially aligned to the maxillary arch model based on a vestibular scan of both models. The face scan with retracted lips was subsequently deleted (as it was only utilized as a template). The result was a model pair that was spatially correctly registered to the complete resting face scan, henceforth referred to as a 3D jaw-face scan (Fig. 1).

### Landmark placement

**Fig. 2:**
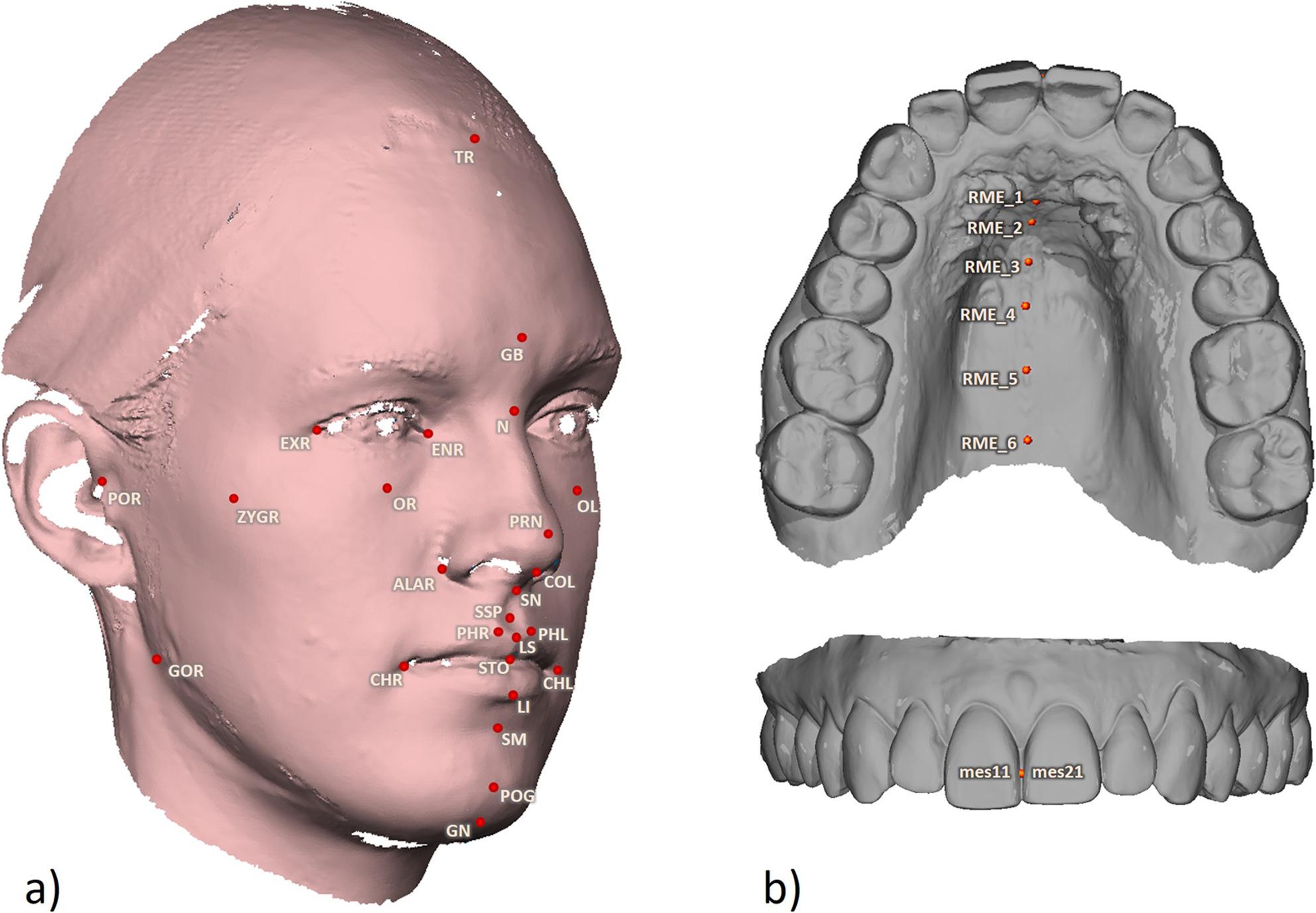
Landmark positions - Landmark positions on a (**a**) 3D face scan and (**b**) virtual jaw model used in the analysis

The 3D jaw-face scans were evaluated using landmark placement via a routine programmed in-house using MATLAB^®^ (version 9.5 R2018b; The MathWorks, Massachusetts, USA). The facial landmarks used (Fig. 2a; Table 1) are based on those described by Farkas [9] and Plooij [8]. On the maxillary models, both dental landmarks and gingival landmarks in the region of the median palatine raphe were selected (Fig. 2b).

**Table 1:** Intra- and interrater variability of anatomical landmarks on facial and intraoral scans as estimated by combining standard deviations with simple linear models; corresponding results were used to calculate 95% confidence intervals

Landmark	Acronym	Intrarater variation [mm] ± 95% CI				Interrater variation [mm] ± 95% CI			
		Transversal (x)	Sagittal (y)	Vertical (z)	3D	Transversal (x)	Sagittal (y)	Vertical (z)	3D
Facial Landmarks									
Nasion	N	0.5 ± 0.07	0.1 ± 0.02	0.7 ± 0.1	0.7 ± 0.1	0.7 ± 0.08	0.1 ± 0.02	1.0 ± 0.12	1.0 ± 0.13
Orbitale right/left	OR	0.9 ± 0.13	0.3 ± 0.05	1.0 ± 0.13	0.9 ± 0.13	1.1 ± 0.14	0.5 ± 0.07	1.5 ± 0.14	1.6 ± 0.15
	OL	0.9 ± 0.13	0.3 ± 0.04	1.0 ± 0.13	0.9 ± 0.11	1.5 ± 0.14	0.5 ± 0.06	1.6 ± 0.14	1.5 ± 0.13
Exocanthion right/left	EXR	0.6 ± 0.1	0.6 ± 0.09	0.5 ± 0.07	0.6 ± 0.10	0.8 ± 0.11	0.7 ± 0.11	0.6 ± 0.09	0.8 ± 0.11
	EXL	0.7 ± 0.1	0.7 ± 0.09	0.4 ± 0.06	0.6 ± 0.09	0.9 ± 0.12	0.8 ± 0.11	0.6 ± 0.06	0.8 ± 0.11
Endocanthion right/left	ENR	0.4 ± 0.06	0.6 ± 0.1	0.4 ± 0.05	0.6 ± 0.08	0.5 ± 0.07	0.7 ± 0.12	0.4 ± 0.06	0.6 ± 0.08
	ENL	0.4 ± 0.05	0.5 ± 0.09	0.4 ± 0.05	0.5 ± 0.06	0.4 ± 0.05	0.6 ± 0.11	0.4 ± 0.05	0.5 ± 0.07
Porion right/left	PR	0.4 ± 0.09	0.8 ± 0.14	0.7 ± 0.1	0.6 ± 0.11	0.7 ± 0.11	1.6 ± 0.16	1.6 ± 0.12	1.3 ± 0.14
	PL	0.4 ± 0.08	0.7 ± 0.13	0.6 ± 0.1	0.5 ± 0.1	0.8 ± 0.12	1.5 ± 0.13	1.4 ± 0.13	1.2 ± 0.12
Labrale superius	LS	0.4 ± 0.05	0.1 ± 0.01	0.3 ± 0.04	0.3 ± 0.03	0.5 ± 0.05	0.1 ± 0.01	0.4 ± 0.04	0.3 ± 0.04
Pronasale	PRN	0.4 ± 0.06	0.1 ± 0.01	0.6 ± 0.09	0.5 ± 0.08	0.5 ± 0.06	0.1 ± 0.02	0.8 ± 0.11	0.7 ± 0.09
Subnasale	SN	0.4 ± 0.05	0.4 ± 0.06	0.4 ± 0.06	0.4 ± 0.06	0.5 ± 0.05	0.6 ± 0.08	0.6 ± 0.07	0.6 ± 0.08
Pogonion	POG	0.7 ± 0.1	0.3 ± 0.08	1.1 ± 0.17	1.1 ± 0.18	1.1 ± 0.11	0.3 ± 0.09	1.3 ± 0.18	1.3 ± 0.2
Gnathion	GN	0.8 ± 0.11	1.2 ± 0.2	1.2 ± 0.19	1.6 ± 0.24	1.2 ± 0.13	1.4 ± 0.18	1.5 ± 0.17	1.9 ± 0.21
Dental Landmarks									
Mesial crown diameter tooth 11/21	mes11	0.1 ± 0.03	0.3 ± 0.06	0.5 ± 0.09	0.3 ± 0.06	0.2 ± 0.04	0.5 ± 0.07	0.7 ± 0.08	0.6 ± 0.07
	mes21	0.1 ± 0.03	0.3 ± 0.05	0.4 ± 0.08	0.3 ± 0.05	0.2 ± 0.04	0.4 ± 0.06	0.7 ± 0.07	0.5 ± 0.07
Landmarks on Raphe palatina mediana (RPM)									
2nd pair of rugae palatinae	RPM_1	0.2 ± 0.03	0.6 ± 0.12	0.9 ± 0.16	0.8 ± 0.15	0.3 ± 0.04	0.7 ± 0.13	1.1 ± 0.17	1.0 ± 0.16
3rd pair of rugae palatinae	RPM_2	0.2 ± 0.03	0.7 ± 0.13	0.8 ± 0.16	1.0 ± 0.17	0.2 ± 0.04	0.9 ± 0.15	1.1 ± 0.17	1.3 ± 0.19
Halfway between RPM_2 and RPM_4	RPM_3	0.2 ± 0.04	1.1 ± 0.16	0.6 ± 0.12	1.2 ± 0.19	0.2 ± 0.04	1.3 ± 0.17	0.7 ± 0.13	1.4 ± 0.2
At height of 2nd premolars	RPM_4	0.3 ± 0.05	1.6 ± 0.21	0.4 ± 0.08	1.6 ± 0.22	0.3 ± 0.05	1.9 ± 0.22	0.5 ± 0.1	1.9 ± 0.23
Halfway between RPM_4 and RPM_6	RPM_5	0.3 ± 0.05	1.3 ± 0.17	0.2 ± 0.16	1.3 ± 0.16	0.3 ± 0.05	1.5 ± 0.18	0.2 ± 0.19	1.4 ± 0.17
Most dorsal point on hard palate	RPM_6	0.3 ± 0.05	1.2 ± 0.18	0.2 ± 0.05	1.1 ± 0.16	0.4 ± 0.05	1.7 ± 0.23	0.3 ± 0.07	1.5 ± 0.21

All landmarks were placed three times on each scan by four examiners in a randomized order and at least one week apart to avoid recall effects. For the face scans, the porion (porion right/left: PR/PL), orbital (orbital right/left: OR/OL), endocanthion (endocanthion right/left: ENR/ENL), exocanthion (exocanthion right/left: EXR/EXL), and nasion (N) landmarks were placed first. The face scans were then spatially oriented using these landmarks to obtain reproducible views of the 3D surface. All other landmarks were selected in a predefined order and with preset views. For the landmark placement on the dental arch models, fissure landmarks of the first molars and premolars were utilized, and the order and views were also standardized. 

### 3D coordinate system

Based on the landmarks placed on the 3D jaw and facial scans, various 3D variables were calculated to characterize the 3D spatial relationships between the dental arch model and facial structures. To calculate these variables, a 3D coordinate system was constructed using three linear equations. Each of the three equations specifies that the position of two landmarks, or the line between these two points, lies on one of the three spatial planes. Specifically, this concerns the following planes and point pairs:

1. the midsagittal plane and the landmarks labrale superius (LS) and Nasion (N);
2. the frontal plane and the center points of the right and left eye (CPR, CPL), which are constructed from the bisector of the lines EXR-ENR and EXL-ENL, respectively; and.
3. the horizontal plane and the landmarks mid-porion (MP) and mid-orbital (MO), which are formed as bisectors of the lines PR-PL and OR-OL, respectively.

In mathematical terms, this means that

1. the basis vector X (transverse) is perpendicular to the line LS-N,
2. the basis vector Y (sagittal) is perpendicular to the line CPR-CPL, and.
3. the basis vector Z (vertical) is perpendicular to the line MP-MO.

**Fig. 3:**
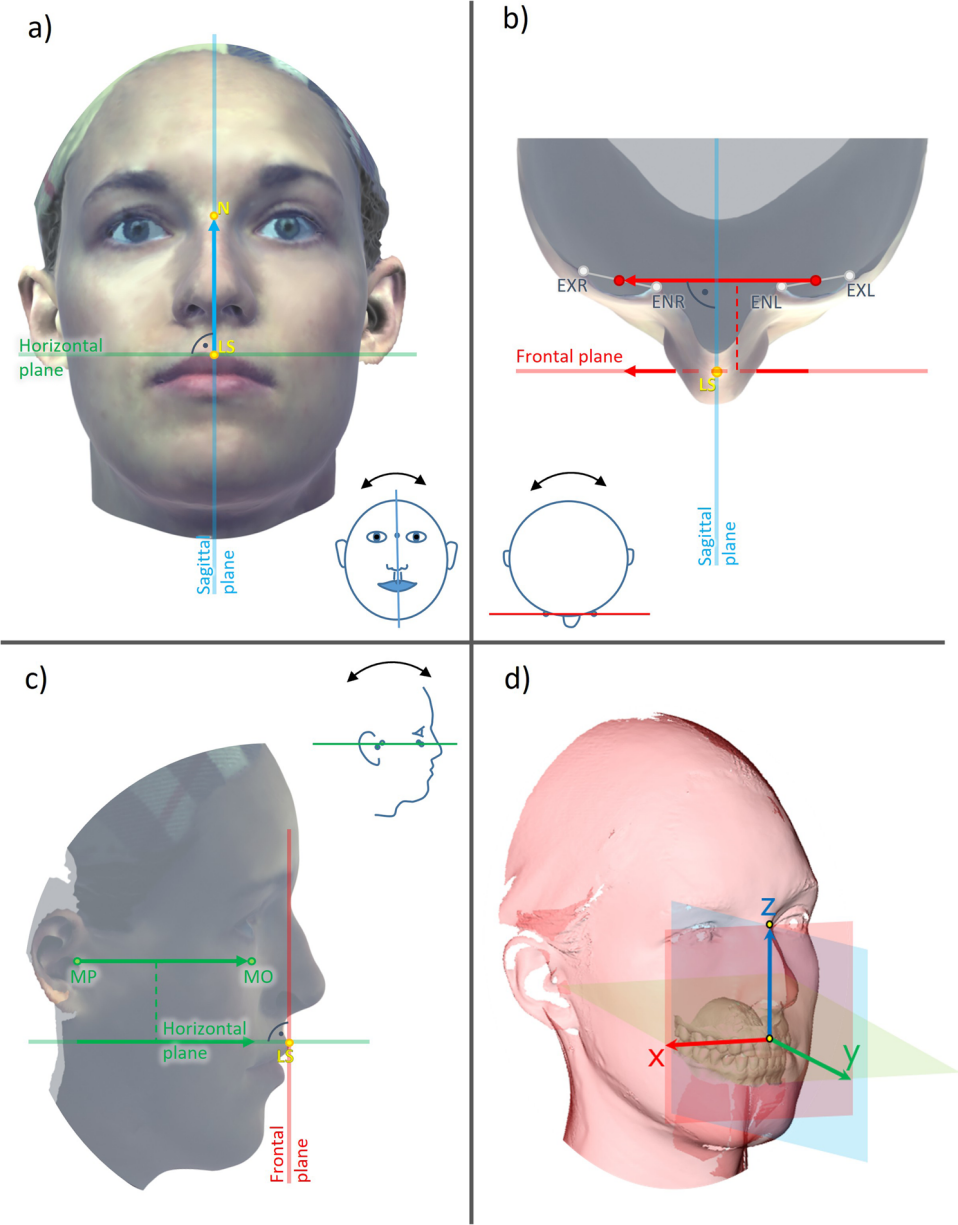
Creation of a 3D coordinate system – Construction of sagittal (**a**), frontal (**b**), and horizontal (**c**) planes for defining a 3D coordinate system originating in LS (**d**). MP and MO refer to geometric midpoints between PR/PL and OR/OL, respectively

According to convention, the basis vectors are orthogonal and normalized. Furthermore, they form a right-handed coordinate system. The origin of the coordinate system’s axes aligned in this way was set to point LS. Consequently, the median, frontal, and transverse facial planes intersect at this point (Fig. 3).

### Dentition-to-face analysis

Facial and dentofacial transverse asymmetries were evaluated based on measurements of the positional relation of specific selected landmarks to defined planes. The facial median plane (facMP) is represented by the sagittal plane, in which the landmarks N and LS lie. The contact point between the upper central incisors (dentMid) was constructed from the established landmarks mes11 and mes21. A plane was fitted through six landmarks in the area of ​​the median palatine raphe (RPM 1–6), representing the median plane of the nasomaxillary complex (maxillary median plane, maxMP). The perpendicular distances of the landmarks pronasale (PRN), pogonion (POG), and gnathion (GN) to facMP, as well as the perpendicular distances of dentMid to facMP and maxMP planes, were calculated. In addition, the distance between the maxMP and facMP planes in the area of ​​the dentMid point was determined. The angular deviation between the facMP and maxMP planes was measured after projection of these planes onto the horizontal plane leading to two intersecting lines.

### Statistical analyses

The variability of landmark placement was evaluated using a pilot study with 10 participants. A mixed linear model was developed using the 3D landmark placement data from 5 raters with 3 repetitions. This multivariate mixed effects model was adjusted to the acquired data in the three spatial axes (x, y and z). The various landmarks on the facial scans were considered separately. The influence of the raters and test subjects was considered as a random effect for each coordinate. The mixed effects model consists of statistical three-dimensional mixed linear models in which correlations between the spatial dimensions were modeled utilizing an unstructructured covariance matrix and random effects for test subjects and raters were taken into account. Based on the estimates of the individual coordinates and the estimated variance (σ² as maximum observed variance between axes), the required number of observations (sample size) for a given confidence interval width (CI; w = 0.25 mm) was determined. The necessary number of observations was calculated using the formula *N* = 4(σ²/w²)z1- σ/2, where z1- σ/2 corresponds to the 1-σ/2 (σ = 0.05) quantile of the standard normal distribution [26]. The required number of observations resulting from the model was then divided according to the ratio of the variances within the groups. This resulted in a minimum of 69 test subjects, which had to be analyzed three times by four raters.

A linear mixed-effect model with data from all included jaw-face scans was used to determine the estimated “true” position of all landmarks (mean coordinate estimated by fixed effect estimates in the regression model) and corresponding 95% CIs from the landmark positions placed by the four raters. The individual influence of each examiner and the respective face being examined were considered as random effects, and the respective landmark was considered as a fixed effect. We estimated the intra-rater variability in landmark positioning separately for the three spatial directions by calculating the standard deviation (SD) values for the results of the three repeats separately for each investigator and scan. These SD values were then combined using a simple linear model to determine overall variation and to calculate 95% CIs. Additionally, landmark positions assigned by different investigators (i.e., inter-rater variability) were estimated separately for the three spatial directions by calculating SD values for the results of all four investigators separately for each repeat and scan. These SD values were then combined using a simple linear model to characterize overall variation. 3D intra-/inter-rater variability was calculated using the formula 3D = √(x^2^ + y^2^ + z^2^), and the linear model was applied to calculate overall variation.

To investigate intra- and interrater variability in the positioning of each landmark separately, the positioned landmarks of all 12 ratings that were available for each participant were placed on the respective scan surface and the deviations from the estimated true position were determined.

To investigate the variability of the values ​​calculated from the landmark positions, a transformation into a common 3D coordinate system was required. To create this system, an averaged 3D coordinate system was calculated from the estimated “true” positions of the 12 individual ratings available for each 3D jaw-face scan. Both, the origin of this common coordinate system and the origin of the 12 individual coordinate systems created from the 12 individual ratings were set at the LS point, and the 3D analysis values ​​and their variability were subsequently calculated.

In patient group A, the calculated values ​​were compared to determine the interdependence of the parameters “deviation of dentMid from facMP” on the one hand and “deviation of maxMP from facMP” on the other hand. For this purpose, median values ​​and 95% CIs were calculated, and the median values ​​were compared pairwise using a Wilcoxon test. Furthermore, the Spearman correlation coefficient was calculated for analysis of the correlation between the two parameters.

## Results

### Deviations between the facial, maxillary, and dental midlines

**Fig. 4:**
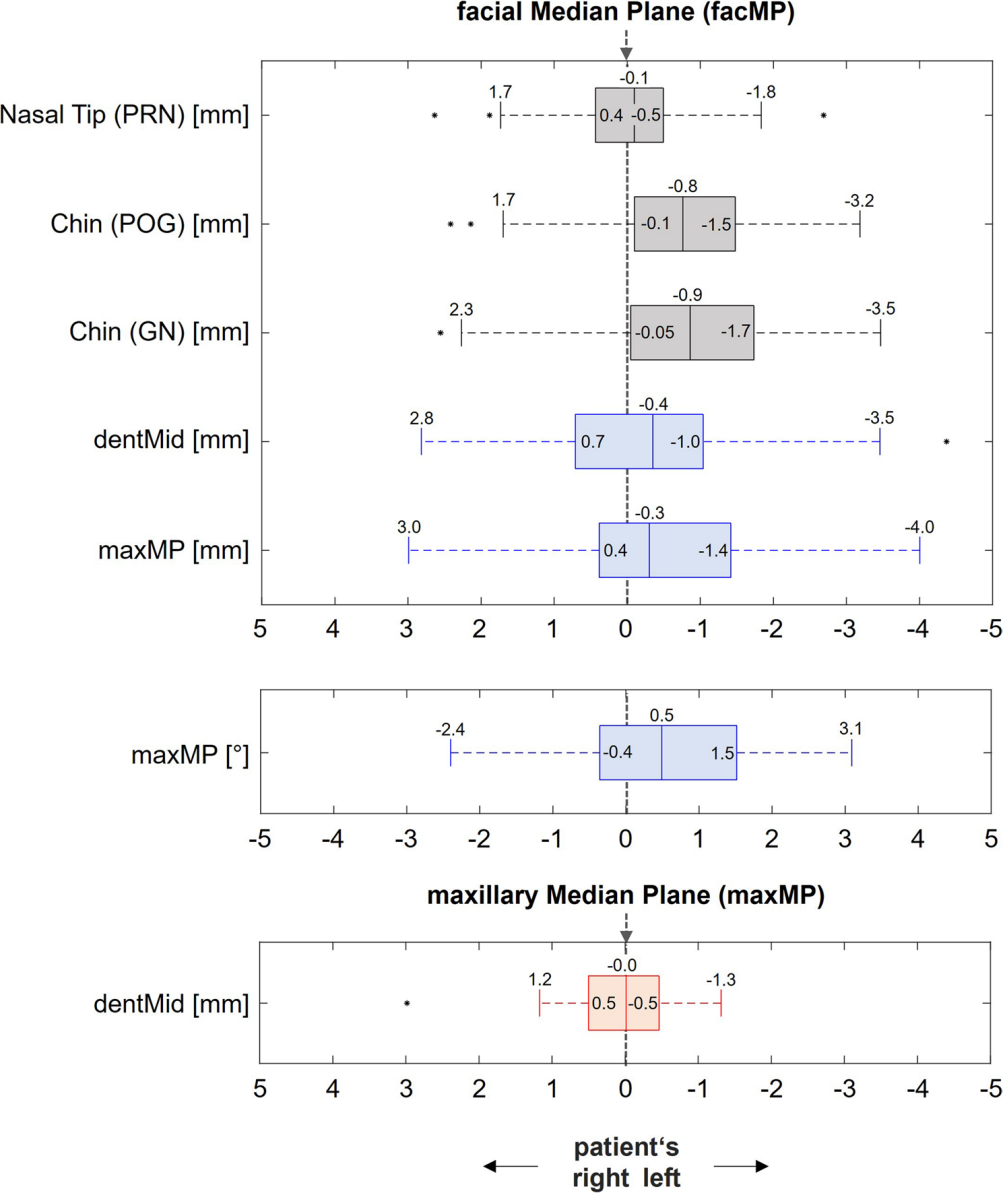
Transverse deviations in patients of group A - Group A included patients without prior orthodontic treatment and without signs of tooth migration in the maxillary anterior region (*n* = 66). Black: facial parameters, blue: dentofacial parameters in relation to facMP, red: dentofacial parameters in relation to maxMP. Upper graph: linear deviations of facial (black boxes) and dentofacial (blue boxes) parameters from the midfacial plane. The distance between maxMP and facMP was measured at the sagittal level of dentMid. Middle graph: angular deviation of maxMP from facMP, projected onto the horizontal plane; due to the use of a right-handed coordinate system, negative signs indicate an angular deviation to the right and positive signs an angular deviation to the left. Lower graph: linear deviation of dentMid from maxMP (red box)

**Fig. 5:**
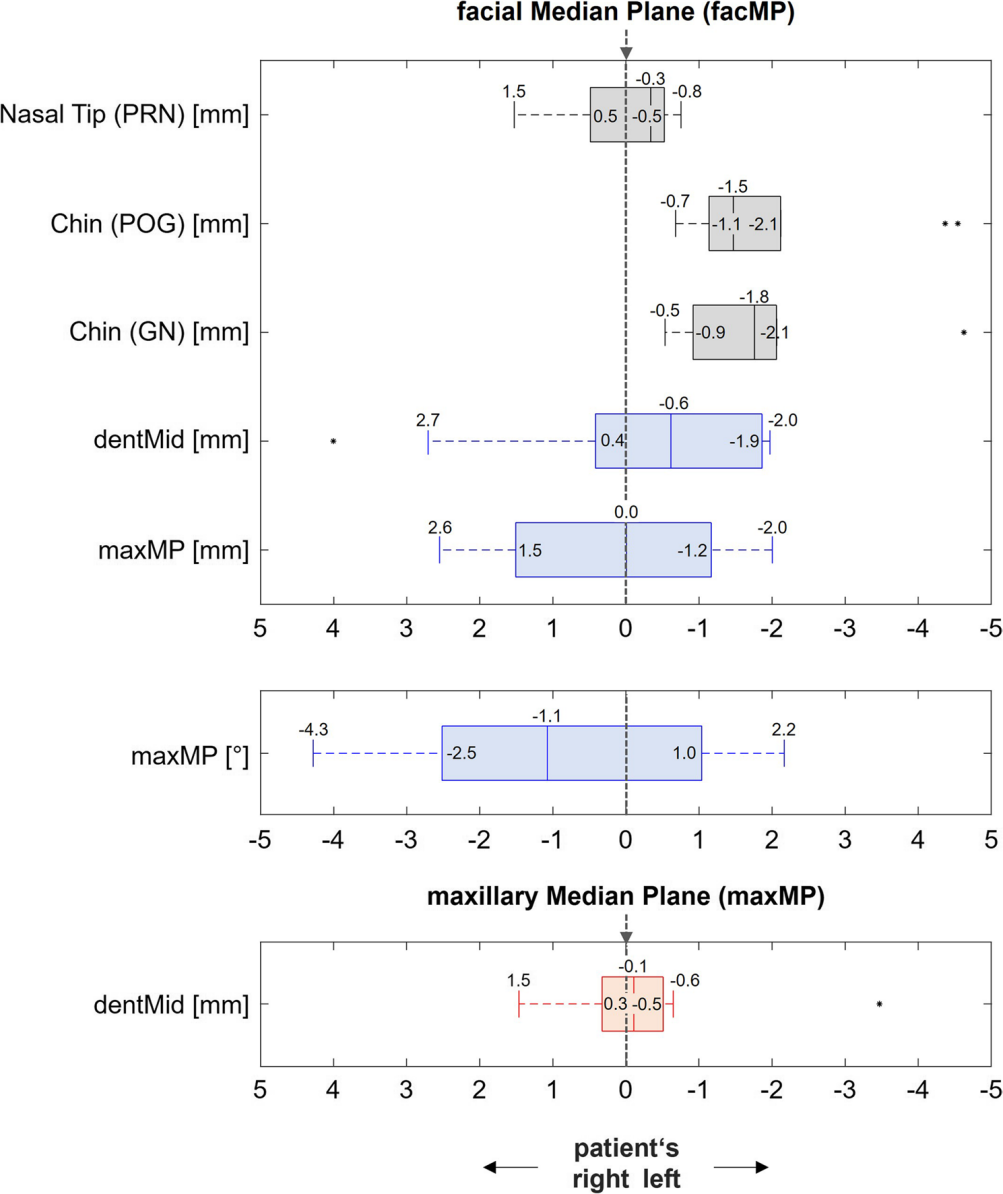
Transverse deviations in patients of group B – Group B included patients without prior orthodontic treatment and with signs of tooth migration in the maxillary anterior region (*n* = 10). Black: facial parameters, blue: dentofacial parameters in relation to facMP, red: dentofacial parameters in relation to maxMP. Upper graph: linear deviations of facial (black boxes) and dentofacial (blue boxes) parameters from the midfacial plane. The distance between the maxMP and facMP was measured at the sagittal level of dentMid. Middle graph: angular deviation of maxMP from facMP projected onto the horizontal plane; due to the use of a right-handed coordinate system, negative signs indicate an angular deviation to the right and positive signs indicate an angular deviation to the left. Lower graph: linear deviation of dentMid from maxMP (red box)

Figures 4 and 5 illustrate the individual results for the 66 patients without frontal tooth migration (Group A) and the 10 patients with detectable tooth migration (Group B), respectively.

In patients without detectable tooth migration (Group A, Fig. 4), deviations of the dentMid from the facMP were observed, with a median deviation of -0.4 mm to the left (inter-quartile range [IQR]: 1.7 mm; range: +2.8 to -3.5 mm). The distance between the maxMP and the facMP measured at the same height showed a median deviation of -0.3 mm to the left (IQR: 1.8 mm; range: +3.0 to -4.0 mm). The angle between the maxMP and the facMP showed a median deviation of + 0.5° anterior to the left (IQR: 1.9°; range: -2.4° to + 3.1°). In addition, median deviation of the dentMid of -0.01 mm to the left of the maxMP was observed in group A (IQR: 1.0 mm; range: 1.2 to -1.3 mm).

In patients with detectable tooth migration (Group B, Fig. 5), the dentMid showed a median deviation of -0.6 mm (IQR: 2.3 mm, range: +2.7 to -2.0 mm) to the left of the facMP. Further, the maxMP did not show deviation from the facMP (median: 0.0 mm, IQR: 2.7 mm, range: +2.6 to -2.0 mm). The angle between the maxMP and the facMP showed a median deviation of -1.1° (IQR: 3.5°, range: +2.2° to -4.3°), indicating an orientation of the maxMP with the anterior part to the right of the facMP. The median deviation of the dentMid from the maxMP was − 0.1 mm (IQR: 0.8 mm, range: +1.5 to -0.6 mm) to the left.

### Transverse deviations of facial landmarks from the median facial plane

In group A, the nasal tip (PRN) had a median distance of -0.1 mm (IQR: 0.9 mm; range: +1.7 to -1.8 mm) to the left of the facMP. In this group, the chin landmark POG showed a shift of -0.8 mm to the left of the facMP (IQR: 1.4 mm; range: +1.7 to -3.2 mm), and the chin landmark GN showed a shift of -0.9 mm to the left of the facMP (IQR: 1.8 mm; range: +2.3 to -3.5 mm).

In group B, the median deviation of PRN from the facMP was − 0.3 mm to the left (IQR: 1.0 mm; range: +1.5 to -8.8 mm). The chin landmark POG deviated by a median of -1.5 mm to the left of the facMP (IQR: 1 mm; range: 0.7 to 2.1 mm), and the chin landmark GN deviated by -1.8 mm to the left of the facMP (IQR: 1.1 mm; range: 0.5 mm to -2.1 mm).

### Correlation between deviations of the dental midline and raphe palatina from the median facial plane

**Fig. 6:**
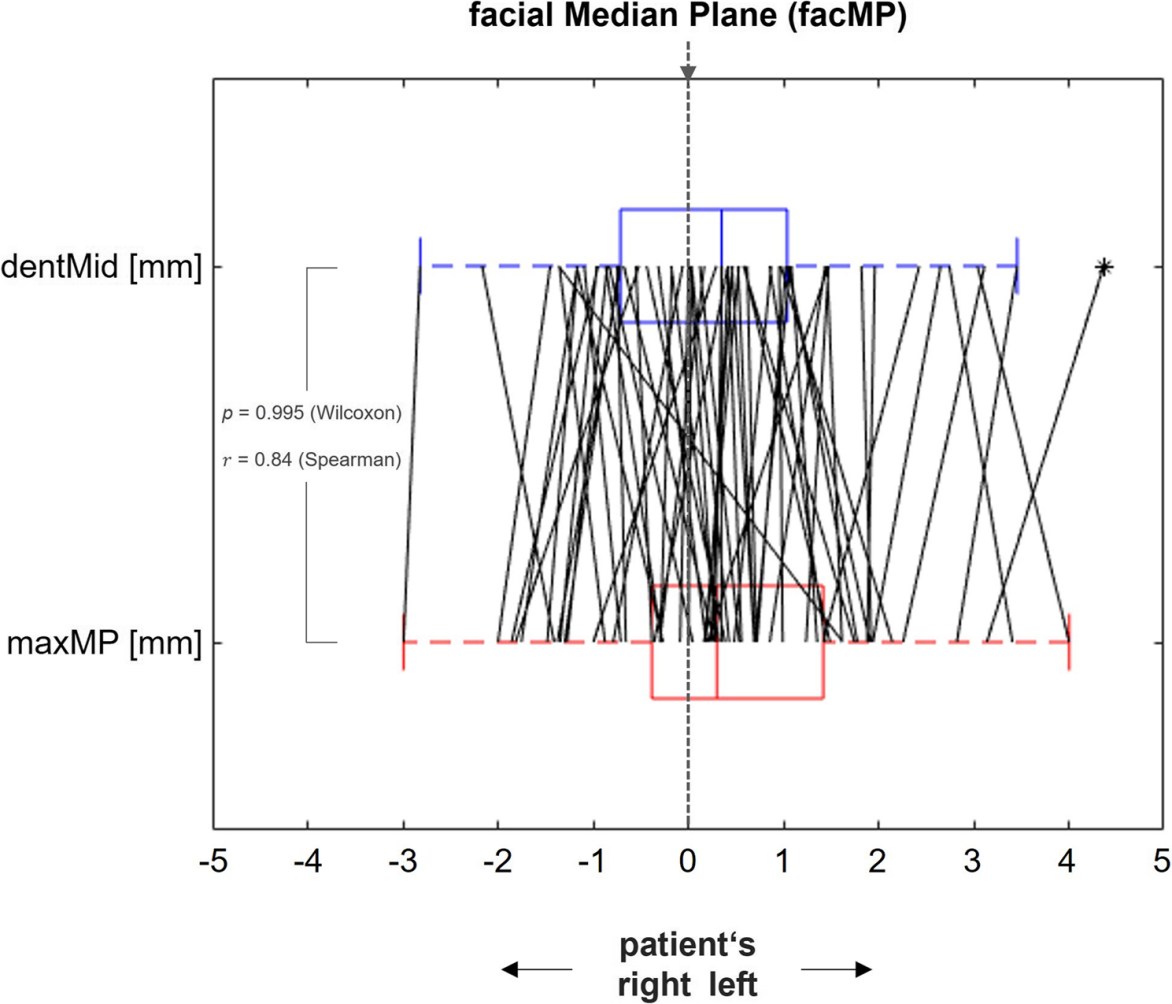
Correlation and pairwise comparison of dentMid–facMP and maxMP–facMP deviations. The analysis was performed in patients without prior orthodontic treatment and without signs of tooth migration in the maxillary region (Group A, *n* = 66)

Figure 6 shows the pairwise correlations between the deviations of the dentMid and the maxMP from the facMP for group A. Pairwise comparison using the Wilcoxon test yielded a very high p-value of 0.995 (95% CI: -0.18 to + 0.17; interval width: 0.35 mm), indicating no significant difference in these deviations. Spearman correlation analysis revealed a correlation coefficient (𝑟) of 0.84, which indicates a strong correlation between the two deviations.

### Intra- and inter-individual variability in landmark placement and analyzed parameters

Mean estimates and CIs for intra- and inter-rater variability in the relevant facial, dental, and gingival landmarks are presented separately for the transversal, sagittal, and vertical planes and for the 3D vector in Table 1. In the transverse direction, the intra-rater variability was less than 1 mm for all facial landmarks, and a maximum of 0.3 mm (CI: ± 0.05 mm) for the dental and gingival landmarks. Further, the inter-rater variability for individual facial landmarks (OR, OL, POG, and GN) was greater than 1 mm in the transverse direction, with a maximum of 1.5 mm (± 0.14 mm) for landmark OL.

Table 2 shows the intra- and inter-rater variability in the deviation values ​​calculated based on the established landmarks. The smallest variability was observed in the distance from the dentMid to the maxMP: the intra-rater variation was 0.3 mm (± 0.05 mm), and the inter-rater variation was 0.4 mm (± 0.05 mm). The largest variability was observed in the measured values of chin deviation at GN, with an intra-rater and inter-rater variation of 1.1 mm (± 0.14 mm) and 1.5 mm (± 0.16 mm), respectively.

**Table 2:** Intra- and Interrater variability of analysis parameters calculated by use of a simple linear model to combine standard deviations of repeats and investigators. Dento-facial and facial parameters indicate deviations from facial median plane (facMP) and maxillary median plan (maxMP), respectively

	Measurement	Intrarater variation ± 95% CI	Interrater variation ± 95% CI
Dento-facial Analyses			
Deviation of upper dental midpoint (dentMid) within face	Distance dentMid – facMP [mm]	0.5 ± 0.06	0.5 ± 0.06
Deviation of dentMid to maxMP	Distance dentMid – maxMP [mm]	0.3 ± 0.05	0.4 ± 0.05
Deviation of maxMP to facMP	Distance maxMP – facMP (upon dentMid level) [mm]	0.6 ± 0.07	0.6 ± 0.07
Rotation of maxMP to facMP	Angle maxMP – facMP (on horizontal plane) [°]	0.9 ± 0.12	1.0 ± 0.12
Facial Analyses			
Deviation of nasal tip	Perpendicular distance PRN – facMP [mm]	0.5 ± 0.07	0.6 ± 0.07
Deviation of the chin	Perpendicular distance POG – facMP [mm]	1.0 ± 0.12	1.3 ± 0.14
	Perpendicular distance GN – facMP [mm]	1.1 ± 0.14	1.5 ± 0.16

To analyze dentofacial midline relationships, the linear and angular relationships between dentMid, facMP, and maxMP were quantified. The corresponding distances of these planes were determined at the level of dentMid. The values ​​for the distance between dentMid and facMP showed an intra- and inter-rater variability of 0.5 mm (± 0.06 mm). The intra-rater and inter-rater variation in the distance between dentMid and maxMP was 0.3 mm (± 0.05 mm) and 0.4 mm (± 0.05 mm), respectively. The distance measurement between the maxMP and facMP planes at level of the dentMid showed an intra- and inter-rater variation of 0.6 mm (± 0.07 mm). The intra- and inter-rater variation for measurement of the angle between the maxMP and facMP planes was 0.9° (± 0.12°) ° and 1.0° (± 0.12°), respectively.

The intra- and inter-rater variabilities for transverse deviation of PRN (the nasal tip) from the facMP were 0.5 mm (± 0.07 mm) and 0.6 mm (± 0.07 mm), respectively. For the deviation of the chin points POG and GN from the facMP, variabilities were larger: intra-rater variation of 1.0 mm (± 0.12 mm) and 1.1 mm (± 0.14 mm), respectively, and inter-rater variation of 1.3 mm (± 0.14 mm) and 1.5 mm (± 0.16 mm), respectively.

## Discussion

The combined 3D face-jaw scan analysis method presented here enables, in this detail for the first time, an objective quantitative assessment of the positional relation between the dentMid to facMP with differentiation of the dental (dental midline shift) and maxillary components (transverse deviation of the raphe maxillae). The findings imply that transverse deviations of the maxillary incisal point can result from skeletal deviations of the nasomaxillary complex and that these can be quantified and differentiated into rotational and translational components. In order to estimate the variability of such deviations in untreated patients without clinically recognizable signs of tooth migration in the maxillary anterior region, these patients were selected from the overall collective (group A). Using these selected patients, the possibility of such a combined face-jaw analysis and its clinical value should be demonstrated.

In the reference group without detectable tooth migration (Group A, *n* = 66), as expected, only negligible average deviations between the dentMid and maxMP were observed (median: -0.01 mm, IQR: +0.5 mm to -0.5 mm) meaning more than 50% of patients showed absolute deviations of more than 0.5 mm. The dentofacial parameters “dentMid to facMP” and “maxMP to facMP” showed more than 50% of absolute values above 0.7 mm–0.4 mm, respectively. Based on these values and taking into account the intra- and interrater variabilities that occur when determining these parameters, a relevant proportion of both the linear and angular values were found to be above the determined intra- or inter-rater variability of the respective parameter. From a clinical perspective, this observation implies that our analysis could help to objectively quantify such deviations in a relevant amount of untreated patients. For smaller deviations, the reliability of the obtained values ​​is likely to be lower, as limitations of the methodology may come into play here: that is, individual differences in landmark placement and errors in merging and aligning the individual scans can add up or cancel each other out. However, it can be assumed that deviations within this methodologically uncertain range have little or no clinical significance, as they lie below the threshold of approximately 2 mm above which deviations of the dentMid from the maxMP have a detrimental effect on dentofacial aesthetics [9–11].

In group B average deviations between the dentMid and maxMP were also small (median: -0.1 mm, IQR: +1.5 mm to -0.6 mm) and the dentofacial parameters “dentMid to facMP” and “maxMP to facMP” showed comparable deviations than in group A (-0.6 mm and 0.0 mm, respectively). Although it might be assumed that dentMid and maxMP correspond more closely in group B, this could not be confirmed and must be investigated and statistically evaluated in studies with larger samples and groups of equal size.

From an aesthetic point of view, the most relevant deviations are the deviations of the maxillary incisal point from the median facial plane. The threshold values ​​above which an aesthetic impairment is perceived are on average 2 mm [10, 11]. These threshold values are lower for assessments made by dentists or orthodontists, but do not go lower than 1 mm [15–18]. The variability for this parameter was 0.5 mm with the present method; thus, clinically or aesthetically relevant deviations of the maxillary midline should be detectable with this method. Of the participants in whom obvious extensive upper midline deviations due to tooth migration were excluded, more than 50% showed a deviation of the dentMid from the facMP of more than 0.5 mm. In this group, deviations of the dentMid could, therefore, be explained by other factors, such as asymmetries of the maxMP. In the present method, the maxMP was constructed using six landmarks located along the RPM between the second pair of palatal folds and the transition to the soft palate. Variabilities of up to 2 mm occurred in the sagittal direction along the raphe. Since the height of the palate changes along the raphe, the sagittal variabilities may also explain the vertical variabilities, which also reached up to 2 mm. However, sagittal and vertical deviations do not influence the construction of the maxMP in the transverse dimension. The crucial observation was that the intra- and inter-rater variabilities were the smallest in the lateral direction at 0.2 to 0.4 mm, which implies a robust detection of the raphe by using the selected landmarks.

In the untreated patient group examined here, the smallest frontal asymmetry was observed in the area of ​​the nasal tip. Here, 50% of the deviations were within 0.5 mm to the right or left. This roughly corresponds to the intra- and inter-rater variability in the measurement of this parameter and means that in 50% of cases, the deviation of the nasal tip cannot be reliably detected by evaluating the face scan using the present method. Since the threshold for aesthetic impairment due to nasal deviations is considered 4 mm based on assessments by laypersons (Silva et al. 2013), this methodological limitation is clinically irrelevant. Similarly, in the case of chin deviations, the greatest variability was observed for deviations of the landmarks POG and GN from the facMP (intra-rater variability of 1.5 mm for the distance between GN and facMP), and in more than 50% of the participants examined, the chin deviation was within this range. However, since chin deviations of less than 6 mm are considered to have little influence on facial aesthetics [9], this variability can also be considered of minor clinical importance.

The quantitative and qualitative differentiation of midline deviations is clinically and therapeutically relevant, especially in cases of larger asymmetries. For instance, a significant deviation of the upper incisal point with a simultaneous deviation of the maxMP from the midline plane of the face would indicate that the cause of the midline shift lies in a skeletal asymmetry of the nasomaxillary complex. This can result from a parallel shift, angular deviation between the maxMP and the facMP, or a combination thereof. In such cases, the aim of therapeutic strategies should be dentoalveolar compensation of this skeletal asymmetry to achieve alignment of the middle of the upper dental arch to the midsagittal plane of the face for aesthetic reasons. If the compensation options are insufficient, or if other skeletal disharmonies exist, a transverse surgical correction of the maxilla may also be indicated. Such different treatment options have serious consequences for the space requirement analysis and thus the entire treatment planning in such cases. Thus, in addition to the jaw model and cephalometric findings, the inclusion of the dentofacial parameters described here could be valuable quantitative basis for determining the preferred treatment option.

Our combined jaw-face analysis, while promising, can be limited by technical inaccuracies and landmark placement errors. Crucial factors for the accuracy of the composite 3D facial jaw scans are a sufficient resolution of the face scans, and the correct alignment of the jaws to the scanned facial surface. While the Breuckmann optoTOP-HE scanner is an industrial scanner and specific validation studies for this exact model in dentistry are rare, the underlying structured light technology is well-validated for facial anthropology [27]. Furthermore, it has been specifically employed in recent research as a high-precision reference standard to validate other body-scanning methods [17] and for rigorous data collection in physical anthropology [28]. Instead of digitized plaster models, intraoral scans can be used for digital diagnostics, which are becoming increasingly common in dental practice. Inaccuracies in plaster models mainly result from plaster expansion [29], whereas the digitization process itself, using desktop scanners (often combined with structured light) is considered very precise, since the dental arch is captured as a whole, which prevents “distortion” of the dental arch. The accuracy of desktop scanners is also reflected by their use in many previous studies (investigating the precision of intraoral scans) as reference method (e.g. [30]). In contrast, full arch models generated based on intraoral scanning require the superposition of multiple tooth segments which is actually considered the main source of inaccuracy [31]. In the discussion of the possible error of any of these two methods for digitization of the dental arches, it has also to be noted that for the direct and indirect approaches, the quantitative deviations from the corresponding situation in-situ are much smaller than the target variables of the present study, i.e., the range of deviations between dentMid and maxMP from facMP. Hence, in the context of this study, we consider the inaccuracies introduced by the digitization method as a minor error.

For the tooth surface-based alignment of jaw models to stereophotogrammetry-based facial scans, 3D deviations of approximately 1 mm (3D positional discrepancy) have been reported in the literature. This can result in a tilt of 3.2° in a constructed occlusal plane and a deviation of approximately 0.4 mm of the dental maxillary center [12]. Codari et al. [13] validated the tooth-based alignment of 3D jaw and face scans using CBCT scans based on linear measurements and found no statistically significant differences between the 3D model integrated via tooth surface superposition and the CBCT scan which per se comprises both the facial and dental surfaces. This finding might be surprising, because these studies did not use matting of the tooth surfaces when creating the face scan with retracted lips, which is different from the approach used in the present study. Actually, Rangel et al. [14] investigated the influence of powder application on the accuracy of integrating jaw models into 3D face scans and found that the use of a matting spray had a statistically significant positive effect. However, they found that the effect was not clinically significant and therefore did not recommend the application of matting spray. In our view, evidence for such conclusion is not entirely convincing and, therefore, this topic seems to require further research.

In general, we based the landmark selection for the constructed 3D coordinate system on symmetry aspects and the orientation of the head in its “natural positon” in order to evaluate facial asymmetries from a “common viewing direction”. Obviously, in such definition of a facial coordinate system it cannot be avoided, that also facial landmarks with higher placement variability (such as “orbitale”) are included. In order to minimize the influence of such variability, the origin of the coordinate system was set at point “Labrale sup.”, i.e., close to the structures under primary consideration. Furthermore, it has to be noted that the impact of a different orientation of the facial coordinate system (within certain limits) on the values for the target variables of this study may not be overestimated.

Individual limitations on the part of both the examiner and the patient could also affect the effectiveness of our combined analysis. Since all face scans examined were evaluated a total of twelve times by four examiners, it can be assumed that the “true value” determined in our study or estimated by the statistical model is very precise. However, in clinical practice, only a single evaluation is usually performed and, therefore, potential variabilities in landmark placement and the analysis parameters calculated thereof must be taken into account.

## Conclusion

Within the described limitations, transverse dentofacial asymmetries with aesthetic implications can be detected, precisely quantified, and differentiated into their dentoalveolar and skeletal components with the help of 3D jaw-face scans, i.e., without the use of X-rays. Importantly, deviations of the dentMid in relation to the facMP caused by skeletal components must be considered in therapy planning, as dentoalveolar compensation and/or surgical correction is required in such cases to ensure a harmonic interrelation between the dentition and the face.

## Data Availability

The datasets generated and analyzed as part of the current study cannot be made available publicly for the sake of protecting patient privacy (as they include 3D scans of the face and jaws that contain identifying information). Pooled numerical datasets with de-identified data are available from the corresponding author on reasonable request.

## Abbreviations

3D: Three–dimensional
ALAR/L: Alare right/left
CBCT: Cone–beam computer tomography
CHR/L: Cheilion right/left
COL: Columella
CPR/L: Center point of the eye right/left
CT: Computer tomography
dentMid: Upper dental midline
ENR/L: Endocanthion right/left
EXR/L: Exocanthion right/left
facMP: Facial median plane
GB: Glabella
GN: Gnathion
IQR: Interquartile range
LI: Labrale inferius
LS: Labrale superius
maxMP: Maxillary median plane
mes11/21: Mesial crown diameter of tooth 11/21
N: Nasion
MO: Mid orbitale
MP: Mid porion
OR/OL: Orbital right/left
PHR/L: Philtrum right/left
POG: Pogonion
PR/L: Porion right/left
PRN: Pronasale
RPM: Raphe palatina mediana
SD: Standard deviation
SM: Submentale
SN: Subnasale
SSP: Subspinale
STO: Stomion
TR: Trichion
ZYGR/L: Zygion right/left
